# Solar spectral management for natural photosynthesis: from photonics designs to potential applications

**DOI:** 10.1186/s40580-022-00327-5

**Published:** 2022-08-05

**Authors:** Lihua Shen, Xiaobo Yin

**Affiliations:** 1 Department of Mechanical Engineering, University of Colorado, Boulder, CO 80309 USA; 2 Materials Science and Engineering Program, University of Colorado, Boulder, CO 80309 USA; 3 Department of Mechanical Engineering, The University of Hong Kong, Hong Kong, China

**Keywords:** Spectral management, Photoconversion, Photosynthesis, Greenhouse, Microalgae, Photobioreactor

## Abstract

Photosynthesis is the most important biological process on Earth that converts solar energy to chemical energy (biomass) using sunlight as the sole energy source. The yield of photosynthesis is highly sensitive to the intensity and spectral components of light received by the photosynthetic organisms. Therefore, photon engineering has the potential to increase photosynthesis. Spectral conversion materials have been proposed for solar spectral management and widely investigated for photosynthesis by modifying the quality of light reaching the organisms since the 1990s. Such spectral conversion materials manage the photon spectrum of light by a photoconversion process, and a primary challenge faced by these materials is increasing their efficiencies. This review focuses on emerging spectral conversion materials for augmenting the photosynthesis of plants and microalgae, with a special emphasis on their fundamental design and potential applications in both greenhouse settings and microalgae cultivation systems. Finally, a discussion about the future perspectives in this field is made to overcome the remaining challenges.

## Introduction

Natural photosynthesis is essential to all life on Earth. Through photosynthesis, organisms such as plants, microalgae, and cyanobacteria convert solar energy into chemical energy (biomass) efficiently using water and CO_2_ [1–3]. Photosynthesis fixes more than 120 billion tons of carbon annually through terrestrial plants alone [4] and the CO_2_ fixation efficiency for microalgae is about 10–50 times higher than terrestrial plants [5, 6]. Photosynthetic organisms have evolved highly efficient light-harvesting systems with a quantum efficiency of more than 90% [7]. Nevertheless, the overall photosynthetic efficiency (conversion of light energy to chemical energy during photosynthesis) is extremely low. The theoretical maximum efficiency of photosynthesis has been estimated to be approximately 12%, but in practice, experimental observations are typically well below this value, around 1% [8–10]. The main reason for such a difference includes such as selective utilization of light by the light-harvesting pigments, excitation energy transfer, respiratory metabolism for maintenance and growth, light-saturated photosynthesis, and so on [11]. Recent advances in the field of genetic engineering could help to improve photosynthetic efficiency [12–14]. For instance, Long et al*.* [15] showed an improvement of photosynthetic efficiency and crop productivity by about 15% in Nicotiana (tobacco) through genetic manipulation of photoprotection. More recently, Farinola and co-workers [16] reported an enhancement in photosynthesis of diatom by in vivo incorporation of an organic dye which acts as an antenna and enhances the light absorption of the diatom. Genetic engineering, however, is still limited to species and much effort is still needed to understand the stability and reliability of genetically modified organisms [17, 18].

Photon management, on the other hand, provides an alternative method of augmenting photosynthesis through spectral matching between the incident light reaching the organisms and the absorption of their light-harvesting pigments [19, 20]. Among many photon management strategies, the use of light-emitting diodes (LEDs) has shown their specific advantages including customization of the emitted light spectrum and high degrees of spatial and temporal control such as light intensity, light period, and so forth [21]. In addition, LEDs enable the selection of specific wavelengths in the lighting spectrum matching the absorption of light-harvesting machinery of photosynthesis [22]. Because of these benefits, LEDs play a variety of roles in horticultural lighting, including use in controlled environment research, lighting for tissue culture, and supplemental and photoperiod lighting for greenhouses [23, 24]. Nevertheless, LEDs are accompanied by huge electricity energy consumption [25]. Instead, solar energy is a form of green energy. However, sunlight with broad spectral distribution makes it inefficiently utilized for the photosynthesis of organisms that have relatively narrow light absorption capability. To improve light utilization efficiency, spectral conversion materials emerge and have been demonstrated as a viable way for spectral photon management through a photoluminescent process. Such materials have found extensive applications ranging from light-harvesting, and solid-state lighting to medical therapy [26–28]. Spectral conversion materials have shown their great potential in the area of natural photosynthesis where they convert the less photosynthetically active light into the most photosynthetically active light reaching the photosynthetic organisms for augmenting their sunlight utilization efficiency and productivity [29–32].

There have been some published papers reviewing the latest optical engineering advances to manage light [19, 20]. These reviews, however, mainly summarize different ways for photon management and pay special attention to the natural photosynthesis of microalgae. Distinct from these published reviews, we here focus on photon management with spectral conversion materials and systematically investigate their recent progress from the point of view of fundamental design and, more importantly, case studies of natural photosynthesis in both plants and microalgae. This review mainly consists of four sections: (1) an overview of spectral conversion materials in terms of their working principle and the Figure-of-Merit, (2) a comprehensive literature review of the case studies examining the effects of spectral conversion materials on plant growth in greenhouse settings, (3) a literature review of recent progress on using spectral conversion materials to augment biomass production of microalgae, and (4) a summary of the perspectives and challenges related to the use of spectral conversion materials for natural photosynthesis, attaining higher efficiencies.

## Selective light utilization for photosynthesis

**Fig. 1:**
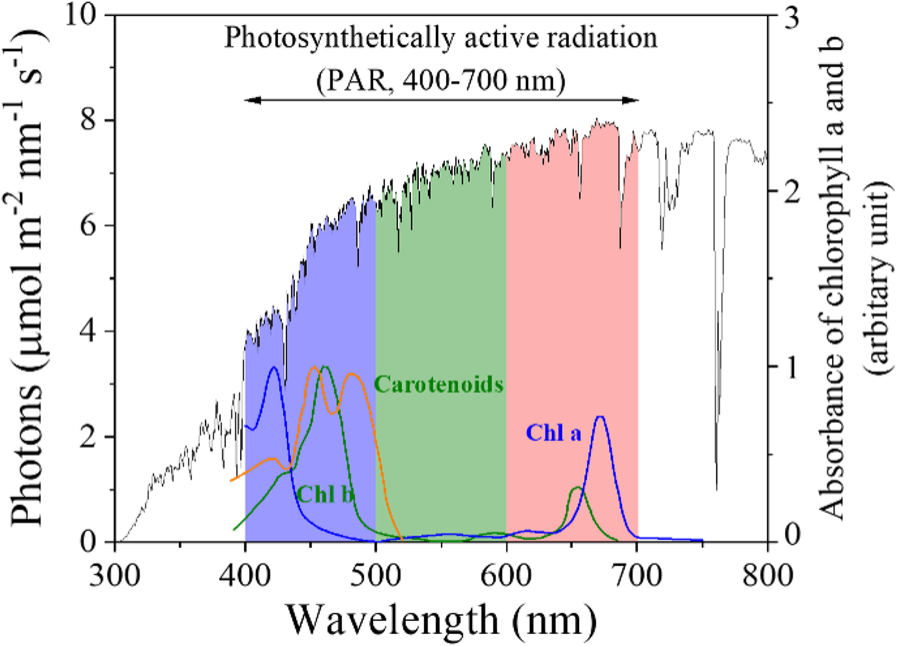
Solar spectral irradiance and absorption spectra of chlorophylls a and b and carotenoids, reproduced with permissions from [33] and [34], respectively

Photosynthesis uses sunlight as the sole energy source. Solar radiation that reaches Earth's surface ranges from ultraviolet to infrared. However, solar radiation between 400 to 700 nm, in general, is considered photosynthetically active (Fig. 1) [35]. The radiation within this wavelength band is also known as the photosynthetically active radiation (PAR), representing approximately 28% and 43% of the solar photons and the total sunlight energy reaching the earth, respectively [20]. PAR is photosynthetically-active because it encompasses the range of wavelengths absorbed by the primary pigments involved in photosynthesis—chlorophyll a and chlorophyll b. These light-harvesting pigments have a spectrally selective absorbance that is high in red (600–700 nm) and blue (400–500 nm) wavebands while low in green (500–600 nm) wavebands (Fig. 1). That is why most photosynthetic organisms appear green. Besides, carotenoids are also ubiquitous and essential pigments in photosynthesis [36]. They have an absorption spectrum mainly between 400 nm and 500 nm and transfer the absorbed energy to the chlorophylls, thus expanding the wavelength range of light driving photosynthesis. Although light responses of the photosynthetic organisms differ based on lighting environment, season, genotype, cultivation practices, and many others, almost all photosynthetic organisms require the same narrow wavelength bands in the blue and red-light region for photosynthesis [37, 38]. Therefore, irradiation of matching the wavelength band for the light-harvesting pigments can maximize the photosynthetic rate.

## Working principle and Figure-of-Merit of spectral conversion materials

**Fig. 2:**
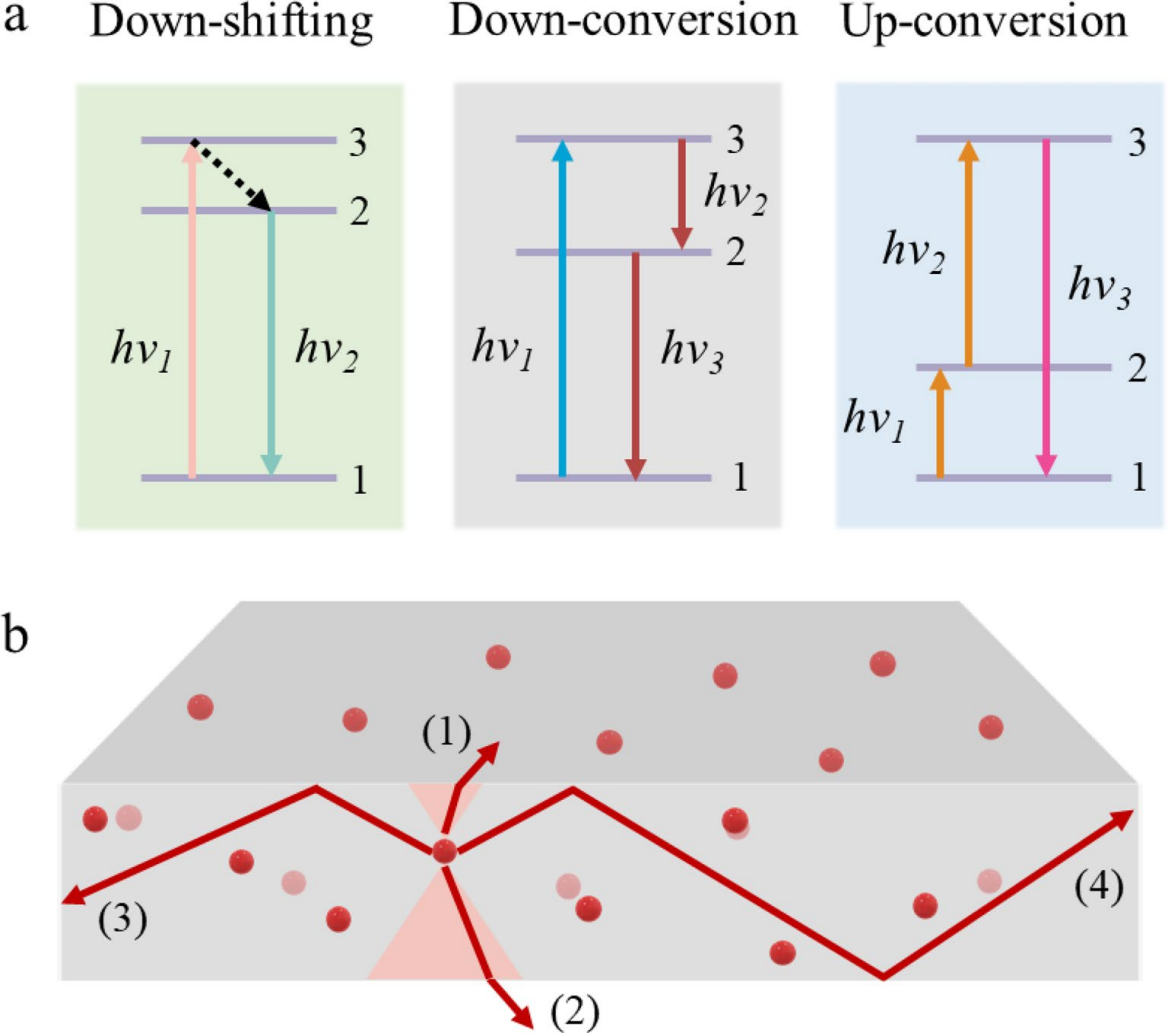
**a** Three photoluminescence processes employed in spectral converters and **b** Schematic illustration of the spectral conversion material with flat designs. The re-emitted photons in the spectral conversion materials either escape from the light escape cone (rays 1 and 2) or are trapped in the materials (rays 3 and 4) due to total internal reflection

Spectrum conversion through photoluminescence has widely been used for augmenting the photosynthesis of organisms by improving the quality of light reaching the organisms [29, 39, 40]. Numerous converters including inorganic phosphors [30, 41], quantum dots [42, 43] and organic fluorophores [39, 44] have shown promising feasibility for spectral conversion of light. Figure 2a presents a schematic illustration of three photoluminescence processes currently under exploration for the development of efficient spectrum conversion materials [19], including down-shifting (DS), downconversion (DC), and upconversion (UC).

The DS and DC materials both involve an optical process of converting high-energy photons to lower-energy ones. Differently, DC converts one high-energy photon into two lower-energy ones, while DS converts one high-energy photon into another photon with lower energy [45]. For this reason, the DC differs from the DS in regard to their quantum efficiency. In the DS process, quantum efficiency will undoubtedly be less than unity (100%), whereas DC has unity or more than unity due to the slight thermal loss [46]. Despite the difference in the spectral conversion mechanism, some researchers use the DC term for DS property materials [47]. Simply, luminescent materials in DS and DC strategy can be categorized into two parts: lanthanides and non-lanthanides. Lanthanides-based luminescent materials rely on luminescent properties of the lanthanide ions which exhibit sharp lines and high efficiency. For instance, Wegh et al*.* demonstrated a downconversion in Eu^3+^-doped LiGdF_4_ with a quantum efficiency close to 200% [48]. In non-lanthanides, a variety of materials are available, mainly including organic dyes, inorganic quantum dots (QDs), and inorganic phosphors. Organic dyes show large absorption coefficients and relatively large photoluminescence quantum yield (QY). For instance, as reported in the literature, Lumogen Red 305 perylene organic dye from BASF shows a QY of 95% in PMMA [49] while 99.6% in polydimethylsiloxane (PDMS) matrix [50]. However, organic dyes generally experience a strong re-absorption loss due to the considerable overlapping between their absorption and emission bands, and usually show poor photostability under solar irradiation [51]. QDs have comparatively wide absorption bands and large emission intensity. In addition, QDs show excellent photochemical stability compared to organic dyes [52]. Even though these advantages, re-absorption loss is still a critical issue in their practical use. Thanks to recent research progress on nanomaterial synthesis, some QDs with nearly zero re-absorption have been synthesized [53, 54]. In contrast, inorganic phosphors show advantages over organic dyes and QDs because of their satisfactory QY, excellent photostability, little re-absorption, and moderate cost [55]. Inorganic phosphors have small absorption coefficients which can be compensated by increasing the concentration of the materials or the thickness of the luminescent layers [46, 56].

Unlike down-shifting and downconversion, upconversion (UC) is an anti-Stokes process that converts low-energy photons, typically in the range of infrared range, into high-energy visible or ultraviolet photons [57]. Since at least two photons must be absorbed in order to create one emitted photon of higher energy than the individual absorbed ones, the internal upconversion quantum efficiency is limited to ≤ 50% [58]. Despite this, photon upconversion is still of interest for applications ranging from luminescence bioimaging [59], and photodynamic therapy [60] to solar energy conversion [61]. Among versatile upconversion materials, the lanthanide-doped upconverters are one of the ideal candidates for controllable and efficient upconversion. Lanthanide-based upconverters generally consist of an inorganic host (mainly heavy halides) and lanthanide dopant ions (e.g. Ho^3+^, Er^3+^, and Tm^3+^) that are dispersed as the guest in the lattice of the host matrix [62]. One representative example is NaYF_4_:Yb^3+^, Er^3+^ based conversion nanocrystals (UCNCs) in which NaYF_4_ serves as a host lattice and Yb^3+^ and Er^3+^ act as a NIR sensitizer and a visible photon emitter, respectively [63]. The abundant and discrete energy levels of lanthanide ions enable multiple emission bands in a broad spectral range from ultraviolet to visible and short-wavelength near-infrared light. Despite the prominent performance such as tunable emission and excellent stability, the lanthanide-based UCNCs still face severe challenges including low quantum efficiency and relatively small absorption cross-section [64].

Another main approach used to achieve upconversion luminescence emission is the so-called triplet–triplet annihilation upconversion (TTA-UC). A typical TTA-UC system is an ensemble of annihilator chromophores (typically polycyclic aromatic hydrocarbons) doped with triplet sensitizer (e.g., metalloporphyrin) [65]. Upon excitation, the sensitizer transfers its triplet energy to the annihilator, followed by the annihilation of two sensitized annihilator triplets, which eventually leads to anti-Stokes delayed fluorescence at higher energy [66]. Compared to the lanthanide-based UC, TTA-UC operates orders of magnitude more efficiently under low-intensity excitation such as white-light illumination and solar radiation due to the broad absorption bands of the organic sensitizer molecules [67, 68]. To date, fundamental theories relevant to each luminescent process are well-established, and there are several in-depth reviews summarizing the key requirements and properties of the most commonly used luminophores for each spectral conversion mechanism [69–71].

For a representative spectral conversion material, it consists of a high optical quality plastic or glass doped or coated by organic or inorganic converters that selectively absorb direct and diffused sunlight and re-emits at different wavelengths (Fig. 2b). The portion of the emitted light that is trapped inside the materials is determined by the refractive index of the materials. According to Snell`s law, all photons approaching an interface between a material and air at an angle higher than the critical angle will be reflected (Fig. 2b, rays 3 and 4). The critical angle is defined as,

1 $${\theta }_{c}={sin}^{-1}\left(1/n\right)$$

where *n* is the refractive index of the materials. For the spectral conversion materials with flat surfaces, these internally reflected photons are trapped in the materials. The trapping efficiency (${\eta }_{trap}$) refers to the fraction of photons re-emitted in the materials trapped via total internal reflection (TIR) [72], which is determined solely by the critical angle,

2 $${\eta }_{trap}=cos\left[arcsin\left(1/n\right)\right]$$

On the other hand, those internally emitted photons at an angle smaller than the critical angle (Fig. 2b, rays 1 and 2) will escape from the spectral conversion materials for external use. Surface emission can be quantified by the external quantum efficiency (${\eta }_{EQE}$), expressed as the ratio of surface emitted photons to all absorbed photons,

3 $${\eta }_{EQE}={\eta }_{QE}\cdot {\eta }_{extraction}\cdot \left(1-{\eta }_{self\_absorption}\right)$$

According to Eq. (3), the ${\eta }_{EQE}$ is dependent on the light extraction efficiency (${\eta }_{extraction}$), the internal quantum efficiency (${\eta }_{QE}$) and the self-absorption efficiency (${\eta }_{self\_absorption}$) of the photoluminescent materials. The ${\eta }_{extraction}$ is defined as the ratio of the surface emitted photons to all internally emitted photons. Both ${\eta }_{QE}$ and ${\eta }_{self\_absorption}$ are essentially determined by intrinsic properties of the photoluminescent materials. Ideally, spectral conversion materials in practical applications should have distinctive characteristics including (1) a high absorption coefficient that enables them to capture enough photons from the incoming light; (2) a high internal quantum efficiency; (3) a minimized attenuation of the internally re-emitted light as they travel out of the materials; (4) a high fraction of the internally re-emitted light extracted in the desired direction towards the organisms; and (5) cost-effective and photostable.

## Spectral conversion materials in agriculture

Photosynthetic organisms selectively use sunlight for photosynthesis and their growth. Converting light with little or no photosynthetic potential to light with higher photosynthetic potential can produce a spectrum that potentially increases the production of photosynthetic organisms. In this perspective, the spectral conversion materials have been widely explored and their potential for photosynthesis and biomass production in photosynthetic organisms including microalgae and plants is further examined. In the PAR region, green light has been considered the least photosynthetically active compared to the blue and red light, with red light being the most efficient for photosynthesis. According to literature, red light is considered ~ 30% more efficient than blue light concerning the photosynthetic action spectrum that shows the relative photosynthetic quantum efficiency per wavelength [34]. Spectral conversion of green to red light paves an indirect way for the use of green light, which accounts for 35% of the PAR. It should be noted that green light could indirectly promote photosynthesis due to its higher penetration [73, 74]. The other important photoconversion process involves the conversion from the non-PAR to PAR, which leads to an increase in the photosynthetic photon flux density (PPFD) and thus potentially augments photosynthesis. Some representative examples include the downconversion of UV radiation to blue [75] or red [76, 77] radiation, and the upconversion of far-red radiation to red [78] or blue radiation [79]. UV radiation usually produces photochemical damage to the cell, reduces photosynthesis, and lowers biomass accumulation of plants [80]. Conversion of UV radiation to PAR could therefore avoid the damage and provide more photosynthetically active radiation, even though in the solar spectrum only less than 2% of the energy falls on UV radiation. Far-red, however, plays an important role in regulating the morphology of the plants during growth, especially when the far red interacts with the red light [81]. The ratio of far-red to red light significantly influences the growth of the plants. In the following sections, we will highlight some practices of such spectral conversion materials for augmenting photosynthesis and biomass production of plants in farming systems.

### Spectral conversion greenhouse claddings

**Fig. 3:**
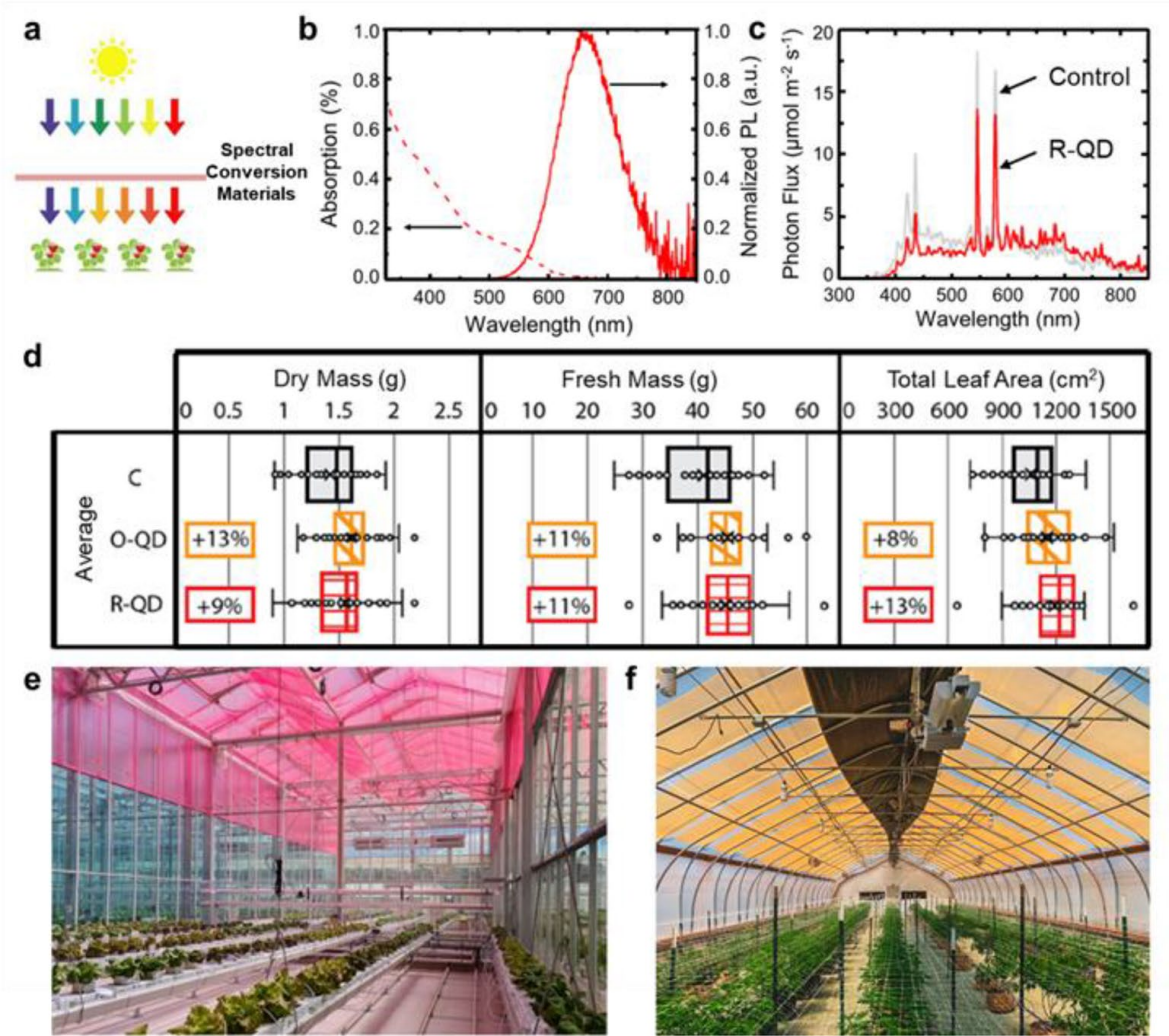
**a** Schematic illustration of spectral conversion materials in a greenhouse setting for enhancing plant growth. **b** Percent absorption and normalized PL emission for CuInS_2_ (CIS)/ZnS quantum dots (QDs)-doped film with peak emissions centered at 660 nm (R-QD). **c** Measured spectra beneath the R-QD film and the control film without QDs. **d** Experimental results of measured edible dry mass, edible fresh mass, and total leaf area for three replicate red romaine lettuce experiments under control film, CuInS_2_ (CIS)/ZnS QDs-doped film with peak emissions centered at 600 nm (O-QD) and R-QD film. b-d are replotted with permission from [43]. **e** Greenhouse covered with the commercially available red LLEAF films for the growth of leafy greens. Republished with permission from LLEAF (http://www.lleaf.com.au/). **f** Greenhouse covered with the commercially available QDs-doped films. Republished with permission from Ubigro (https://ubigro.com)

Using spectral conversion materials as the greenhouse claddings have been proposed since the 1990s [82]. Such spectral conversion materials mainly alter the quality of light that enters the greenhouse interior space for plant growth through a photoconversion process (Fig. 3a). Due to its potential for augmenting biomass productivity of crops in the greenhouse, this technology is still receiving notable attention [83–86]. For example, Bergren et al. demonstrated the benefits of quantum dots (QDs)-based luminescent film technology to improve spectral quality for plant growth [43]. The film consists of CuInS_2_ (CIS)/ZnS QDs with peak emissions centered at 660 nm (Fig. 3b). CIS/ZnS QDs exhibit a large Stokes shift, allowing for absorption in the UV and blue while emitting in the red, which minimizes re-absorption and downconverts the spectrum to more photosynthetically efficient one. For this benefit, the CIS/ZnS QDs-based agriculture films enable passive modification of the incident light (Fig. 3c). Replicated growth experiments demonstrated that dry mass, fresh mass, and total leaf area were increased on average by 9%, 11%, and 13%, respectively, under the CIS/ZnS QDs-doped film with peak emissions centered at 660 nm (R-QD) compared to the control film without QDs (Fig. 3d). Son et al*.* reported a green-to-red spectrum conversion film for the growth and fruit quality of strawberries (*Fragaria* × *ananassa* Duch. cv. Seolhyang) [87]. The spectral conversion polyethylene film led to higher photosynthetic activity over a long time, while it did not change the photosynthetic chlorophyll content or leaf optical properties. Strawberry fruit qualities of such as fruit weight, sweetness, sweet/acid ratio, and firmness were promoted under the spectral conversion film. To date, numerous spectral conversion materials have been explored and investigated for plant growth, depending on the luminescent properties of materials and the plant species, such as Eu^3+^-modified cellulose acetate film [88], CdZnSe QDs-based fluoropolymer film [89, 90], Sr_2_Si_5_N_8_:Eu_2_t-based cellulose film [91], upconversion nanoparticles-based polymer film [78], organic dye-containing polyethylene/ polymethylmethacrylate (PMMA) film [92, 93], and so forth.

Thanks to rapid progress in the development of spectral converters, some products relevant to spectral conversion greenhouse claddings are emerging in the market. One example is the so-called LLEAF 620 film (Fig. 3e) that is fabricated by an Australian company LLEAF [94]. The film is made by incorporating organic fluorescent dye into polycarbonate and these films can be easily retrofitted to existing greenhouse structures by screwing, clamping, or hanging (Fig. 3e). The experimental observations show an increase in the yield of 3 varieties of lettuce. The other commercially available product in the market is an orange film (Fig. 3f) made by a company called Ubigro in the US [95]. The film contains nontoxic luminescent quantum dots and can convert UV and blue light into red light. Trial experiments conducted in North America and Europe show increased yields on crops like tomatoes, cucumbers, cannabis, and some leafy greens. It should be noted that these cladding materials usually have flat designs in the form of films. According to the above discussion in the section entitled “Working principle and Figure-of-Merit of spectral conversion materials” and Fig. 2, a major problem is that only a small portion of the spectrally generated emitted light in the light escape cone can escape from the film [96]. In other words, a major part of the spectrally generated emitted light is still confined due to total internal reflection and eventually is wasted.

**Fig. 4:**
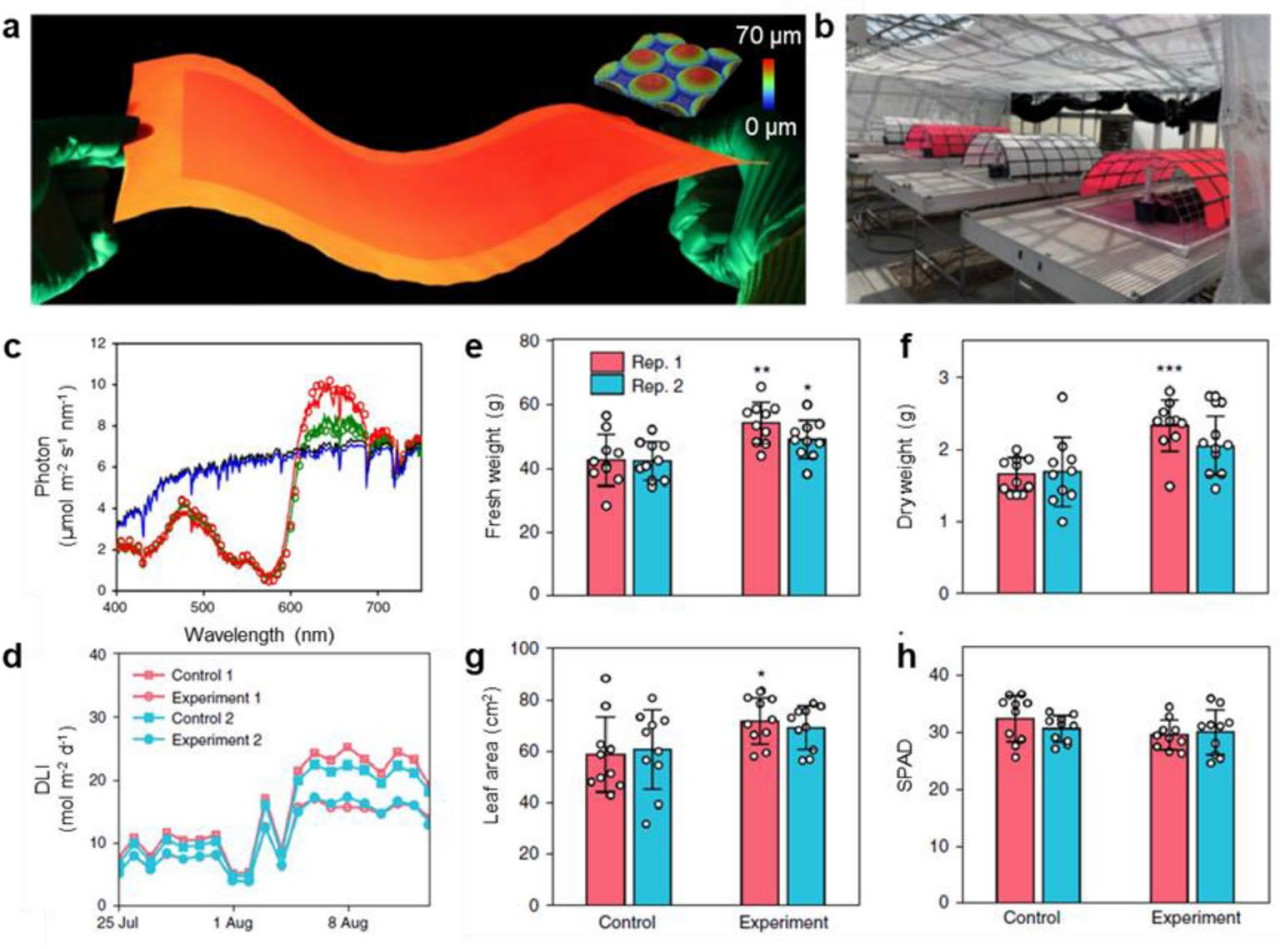
**a** Photograph of a green-to-red spectral conversion film with micro-structures on the top surface under green light illumination. Inset is the surface topography of the micro-structures. **b** Photograph of the semicylindrical roof domes arranged in the greenhouse facility. **c** The forward spectral irradiance of the spectral conversion film with surface micro-structures (red curve), the fluorescent film without surface micro-structures (green curve), the planar fluorophore-free film (black curve), and the fluorophore-free film with surface micro-structures (blue curve) under the emulated solar irradiance. **d** DLI inside all four domes. **e**–**h** Aboveground fresh weight (**e**) and dry weight (**f**), average leaf area (**g**) and SPAD values (**h**) of the lettuces at day 20 after transplantation. Republished with permission from [98]

The key to addressing the confinement of the spectrally generated emitted light is to reduce or even eliminate the total internal reflection and increase the light extraction efficiency. Recently proposed microphotonic structures [97, 98] can effectively extract such internally emitted light and, more importantly, re-direct them into the direction facing the plants for their photosynthesis. The microphotonic structures consist of the well-designed micro-domes which are closely packed on a square lattice on the top surface of the LF305-doped poly(methyl methacrylate) (PMMA) film (inset in Fig. 4a). The strong light extraction effect due to the photon recycling process can be readily recognized visually by the brightness of the central region (Fig. 4a) compared to that of the surrounding areas in which no structures are fabricated. The developed LF305-doped PMMA film with microphotonic structures can be easily used as the greenhouse covering materials for improving plant growth (Fig. 4b). Quantitative analysis shows that the microphotonic film has a high light extraction efficiency of up to ~ 89% and, more importantly, 73% of the externally extracted light is redirected in the direction facing the plants. As a result, the external quantum efficiency of the LF305-doped PMMA film with microphotonic structures increases to around 44%, compared to that of the control film of 18% (Fig. 4c). The authors experimentally demonstrated in the research greenhouse that, though less daily light integral (DLI) under the microphotonic film (Fig. 4d), the biomass production of lettuce is increased by more than 20% without losing the quality (Figs. 4e-h). In addition, nano- or micro-sized scattering particles such as TiO_2_ and ZnO embedded in fluorescent matrices also show potential to improve light extraction efficiency [92, 99]. Embedding scatterers to a greenhouse covering material yields a higher growth performance of crops. However, the light extraction enhancement by these scattering particles is still unknown.

### Fluorescent reflectors in vertical farming

**Fig. 5:**
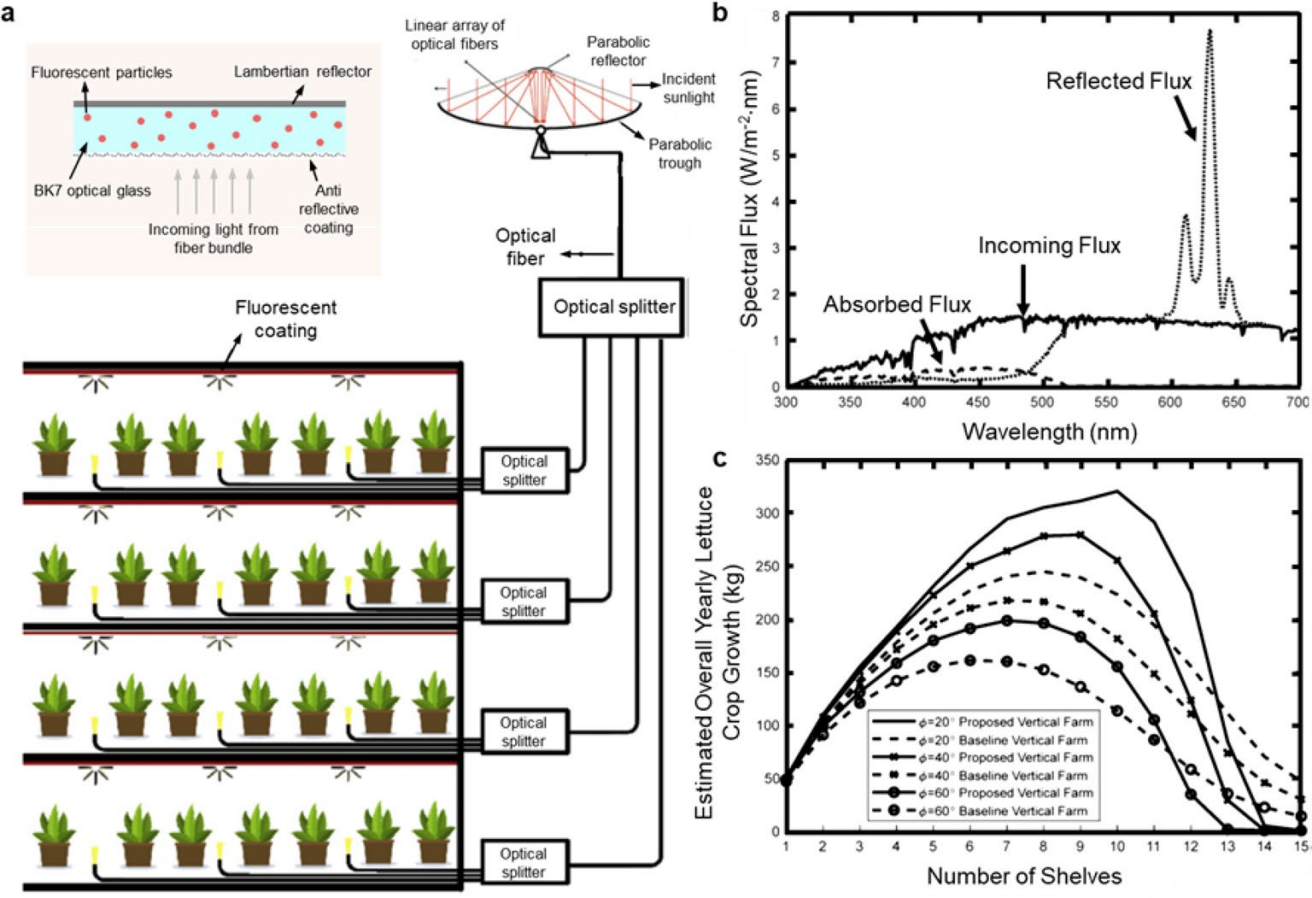
**a** Layout of a vertical farm with a spectral conversion reflector which consists of an optical glass embedded with fluorescent pigments to convert blue-green light to red. **b** Spectral heat flux and reflectance of the coating. **c** Estimated overall yearly lettuce crop growth per greenhouse unit for different shelves number and latitudes, with and without the fluorescent reflector (based on simulations). Republished with permission from [100]

In the case of a polymer-based spectral conversion film with n = 1.5, only less than 13% of the spectrally generated emitted photons can escape equally from the film respectively in the front and backside, according to Eq. 2. In theory, recycling the otherwise wasted light escaping from the backside will increase the emission from the front side facing the plants and double the efficiency. In this view, Erturk et al*.* [100] proposed a fluorescent reflector that comprises optical glass embedded with fluorescent pigments and a Lambertian back reflector (Fig. 5a). The Lambertian back reflector randomizes the direction of the reflected light. Randomizing the direction of light allows much of the reflected light to be internally reflected. Light reaching the front surface at an angle smaller than the critical angle escapes to the air for plant growth. In this specific case, sunlight is collected by a parabolic through the collector and is transmitted into vertical shelves by splitters and optical fibers. The optical fibers direct the sunlight to the fluorescent coatings where the blue-green light is converted to red light. The back reflector recycles the internally generated emitted red photons and enhances the fluorescent emission on the front surface. Attributed to the unique design, spectral conversion from 300–520 nm to 600–650 nm results in a spectral reflectance greater than unity between 600 and 650 nm (Fig. 5b), enabling more photosynthetically active light for crop growth. Simulation results show an overall crop growth increases up to 31% using the fluorescent reflectors (Fig. 5c). The increasing efficiency, however, is dramatically dependent on both light distribution and effective reflectance. Experimental observations show an increase in crop mass of lettuce by about 35% depending on latitude. It should be noted that the Lambertian back reflector in the fluorescent coating might increase the possibility of re-absorption by the fluorescent pigments due to the increased pathlength of the light [101], thus decreasing the overall efficiency of the fluorescent coatings.

### Fiber-coupled luminescent concentrators

**Fig. 6:**
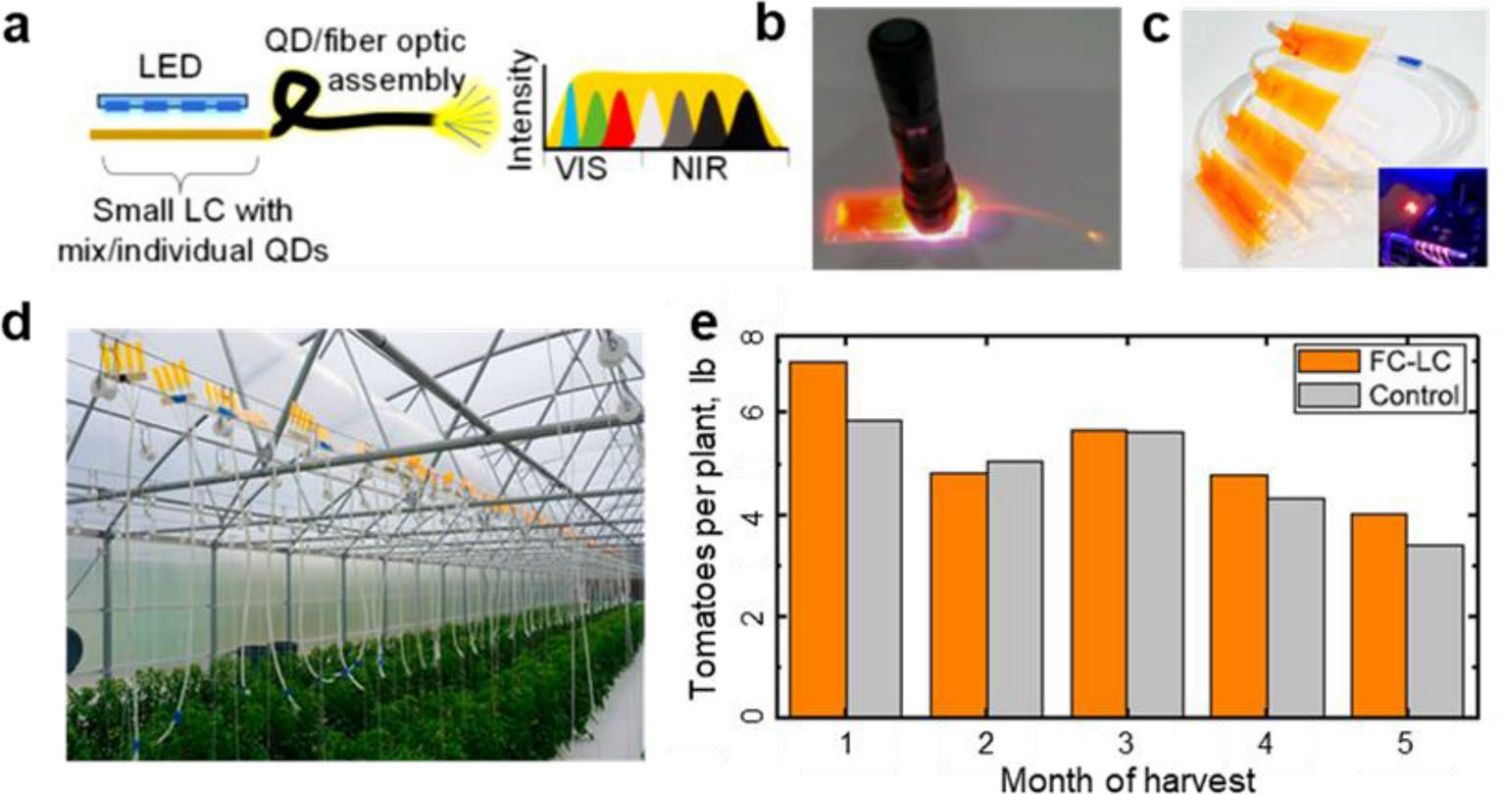
**a** Schematic illustration of a fiber-coupled broadband light source. Quantum dots (QDs) are incorporated into small fiber-coupled luminescent concentrators (FC-LCs). **b** Photographs of a fiber-coupled luminescent concentrator with peak emission at 590 excited by a blue LED flashlight. **c** Photographs of the prototype FC-LCs devices for agricultural deployment. The inset depicts emissions during exposure to large-area blue LED lighting. **d** 82 collectors arrayed over a row of tomato plants in a commercial greenhouse. **e** Total harvested tomato weight (per plant) each month from the plants grown with and without FC-LCs. Reproduced with permission from [102]

Conversely, luminescent solar concentrators (LSCs) in the greenhouse take advantage of the trapped light [56]. Makarov et al*.* developed the fiber-coupled luminescent concentrators (FC-LCs) for low canopy lighting [102]. The luminescent concentrators with CuInS_2_/ZnS QDs emitting at 600 nm absorb the irradiated light from a larger area, re-emit it at different wavelengths, and concentrate the internally emitted light onto an edge with a much smaller area due to total internal reflection (Fig. 6a). In the form of long optical fibers, the internally confined light will transport along the fibers and partly escape from their ends (Fig. 6b). Figure 6c shows multiple prototype fiber-coupled luminescent concentrators consisting of four collectors with five 4 ft-long fibers per collector. The collectors with relatively large surface areas convert the blue portion of the impinging solar spectrum into the orange-red light and the re-emitted light can be then coupled into fiber optics and guided to their ends. In this way, the internally emitted light is transformed from the top canopy to the low canopy. It is worth noting that at low canopy levels under low light conditions, 1% more PPFD results in 0.5–1% crop yield improvement even though the yield improvement also depends on the type of plant, season, temperature, and other growth factors [103]. In a commercial greenhouse (Fig. 6d), these fiber-coupled luminescent concentrators above the plants can direct the re-emitted light to the lower canopy, especially for the crops with tall vines, for instance, tomatoes. Due to the increased light intensity at the low canopy levels, the fiber-coupled luminescent concentrators lead to a 7% improvement in the weight yield of beefsteak tomatoes in the hydroponic greenhouse compared to the control (Fig. 6e). However, such fiber-coupled luminescent concentrators still have a limited efficiency for light delivery. Even though at noon and a clear sunny day, the low canopy only receives around 10% of the PPFD as the upper canopy. The very low efficiency could be improved by reducing surface loss of the internally generated photons from the light escape cone and enhancing the emission at the ends of the optical fibers. Surface loss is key in the luminescent solar concentrators [104] and in the last decades, numerous efforts have been made to minimize the surface loss by aligning the luminophores [105, 106] and using selective mirrors [107–109]. Emission enhancement, on the other hand, can be improved by increasing the light extraction efficiency of the optical fibers. Various strategies have been developed to date to increase light extraction efficiency such as surface roughness [110, 111], graded refractive index materials [112], corrugated structures [113], nano- or micro-structures [114, 115], and so on.

### Greenhouse-integrated wavelength selective photovoltaic panels

**Fig. 7:**
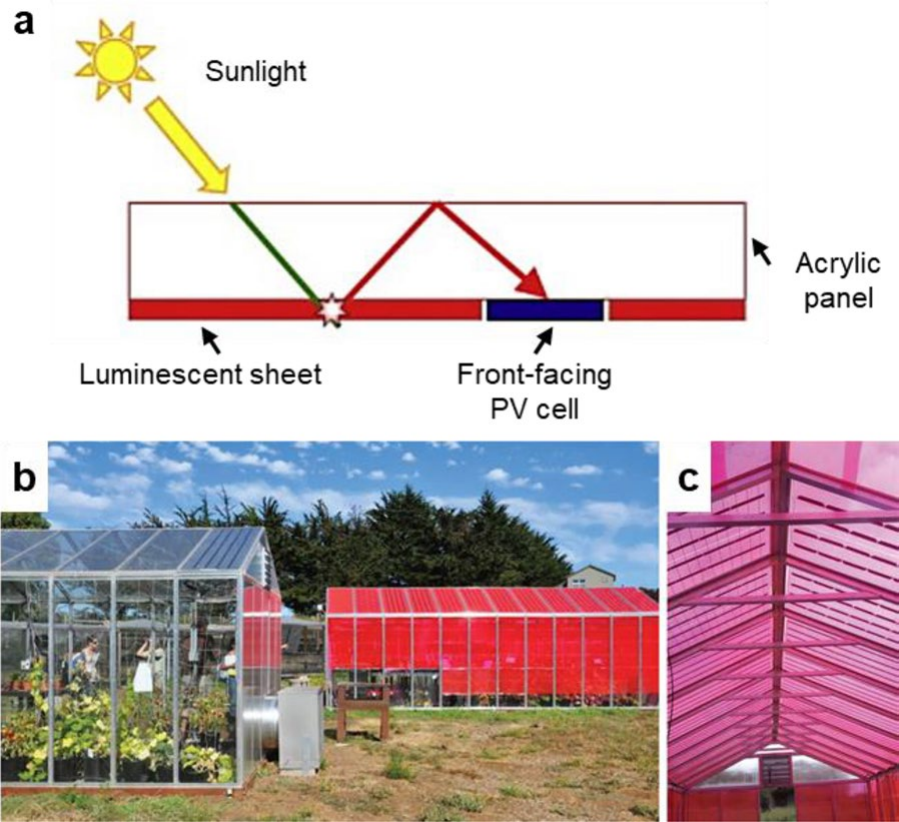
**a** Schematic illustration of a wavelength selective photovoltaic panel with bottom-mounted solar cells. **b** Greenhouse constructed with wavelength selective photovoltaic panels (red colors) at the University of California Santa Cruz Arboretum. **c** Photograph of the greenhouse roof with narrow photovoltaic strips (black lines in red panels) for electricity generation. Reproduced with permission from [118]

The combination of LSCs with photovoltaics (PVs) in greenhouses has also attracted much recent attention [32, 116]. The LSCs with solar cells attached to the greenhouse roof spectrally convert the incident sunlight for plant growth and in the meantime collect the otherwise wasted light for electricity generation. The majority of the LSCs designs place solar cells on the edges of waveguides to maximize light gain. Besides, the bottom-mounted designs have been also systematically investigated in the past years [117]. By orienting the solar cells to face the sunlight, they collect both direct light and concentrated light (Fig. 7a). In addition, for LSCs with significant distances between edges, bottom-mounted cells show benefits in minimizing the average distance traveled by concentrated light in the waveguide. Corrado et al*.* [118] constructed the LSCs panels in the greenhouse which have 13.9% of the back surface covered by the bottom-mounted 20% PV cells (Fig. 7b and c). The use of the LSCs panels exhibits a 9–37% increase in power production compared to the reference, depending on cell alignment and positioning. Initial plant trial results have shown neutral to positive effects on microalgae growth underneath the bottom-mounted LSCs panels in the greenhouse [119]. A follow-up study concluded that these greenhouse LSCs panels had no negative impacts on tomato and cucumber production and that some varieties of crops have slightly higher values of fruit number and mass [79]. Similar LSCs panels that offer wavelength-selective power production have since entered the marketplace, and there appears to be a growing market for the wavelength selective photovoltaic panels that can generate electricity and growth crops simultaneously [120]. Numerous questions, however, remain about the impacts of the light environment underneath such LSCs panels on plant growth. Characterizing the potential impacts of these panels on growth across the huge diversity of crop species should be a high priority for future research.

## Spectral conversion materials for microalgae cultures

Algal biomass serves as valuable feedstocks for biodiesel production and value-added biochemicals [121–123]. Light is the most significant factor governing the entire process of microalgal cultivation in a growth medium (water supplemented with nutrients). The strategies for microalgal cultivations reach from open ponds to photobioreactors and have attracted much attention in the past decade [124–126]. Photon management through spectral conversion materials improves the quality of light reaching the microalgae, thus altering their photosynthesis and biomass production [20, 127]. Considering the difference in microalgal cultivations between open ponds and photobioreactors, the design of spectral conversion materials varies in practice.

### Spectral conversion materials for microalgae cultivation in photobioreactors

**Fig. 8:**
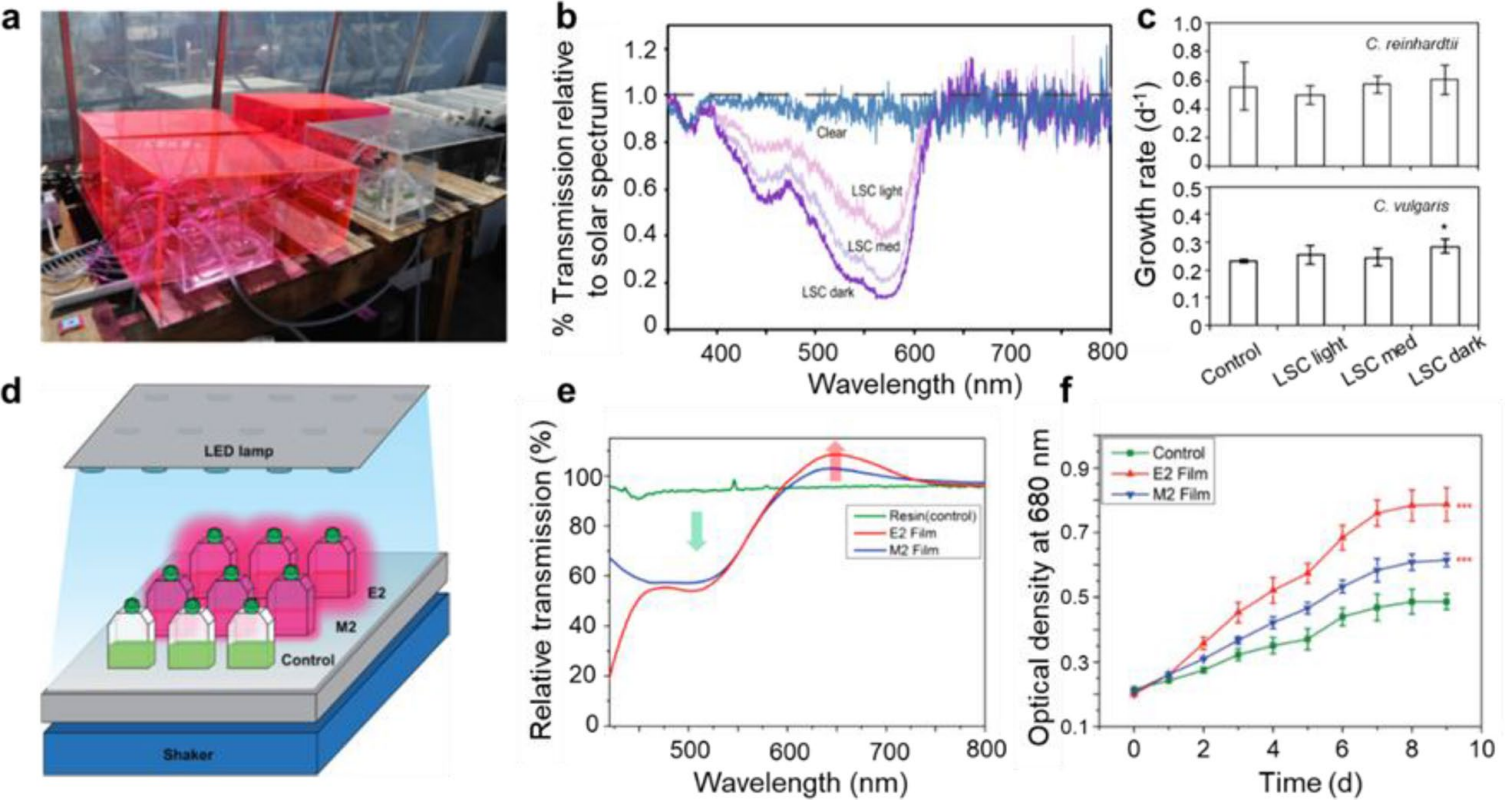
**a** Photograph of microalgae in photobioreactors that were placed in water baths underneath spectral conversion panels and a clear panel. **b** Transmission spectra (350–800 nm) for the three LSC panels and the control panel relative to the solar spectrum recorded outside on a clear day. **c** Growth rates are represented by bar graphs for *C. reinhardtii* and *C. vulgaris* algae. Reproduced with permission [82]. **d** Schematic method of fabricating the photobioreactor for algae cultures and schematic experimental setup of microalgal cultivation. **e** Transmittance change of thin films coated on the culture flasks. **f** Growth of *Chlorella sp.* grown under different cultural conditions. Reproduced with permission from [130]

Compared to open ponds, photobioreactors could significantly minimize the likelihood of contamination by other microorganisms or alien microalgae species and have an improved light utilization efficiency of microalgae due to their geometric designs [128, 129]. When a spectral conversion material is integrated with the photobioreactor, two designs are frequently employed, that is, the front-light design and the back-light design. For example, Prufert-Bebout et al*.* [82] proposed implementation with acrylic panels containing a fluorescent dye, Lumogen Red 305, into growth chambers (Fig. 8a). Such a roofing architecture shows a front-light design where incoming light is partly converted by the spectral conversion materials and then both the non-converted and converted light travel in the air before entering the photobioreactor for microalgae growth. The fluorescent panels mainly absorb green light and emit slightly red light because of light trapping (Fig. 8b). Following experimental tests on different strains of microalgae, no significant impact on algal growth was observed (Fig. 8c). Recently, Kim and coworkers [130, 131] reported the other typical front-light design by directly coating the spectral conversion materials into the photobioreactor. It is schematically depicted in Fig. 8d. The thin films coated on the culture flasks show notable conversion of the spectrum (Fig. 8e). In this case, the front-light design with spectral conversion coatings increased the total lipid production of Chlorella sp. by more than 60% (Fig. 8f). While some observations show improvement in microalgal growth, photons from the spectral conversion materials in the front-light design have no preferential emission direction, and thus a large fraction of photons does not enter the photobioreactor for algae growth, especially for the roofing design. In addition, the spectral conversion materials must be highly transparent for light that are not to be converted.

**Fig. 9:**
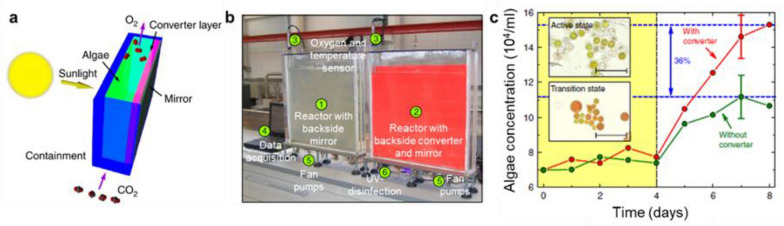
**a** Photobioreactor that is combined with a solar spectral converter in backlight geometry. **b** Photograph of the flat panel photobioreactor set-up. **c** Algae concentration as a function of time. Reproduced with permission from [132].

Back-light design, on the other hand, refers to the spectral conversion materials positioned behind the photobioreactors [132]. Incoming light passes through the photobioreactor and only the unabsorbed light participates in the spectral conversion process. In this design, a reflector is usually used to recycle all transmitted and converted light back towards the algae cultures in the photobioreactor. An example of this approach is schematically shown in Fig. 9a, where a photoluminescent phosphor was coated on a mirror back-plat. Long-term algae proliferation experiments show that the backlight photobioreactor results in 36% more biomass production of *H. Pluvialis* (Fig. 9c). It can be expected that this design is useful only for dilute algae cultures where a meaningful amount of light can reach the spectral conversion materials. Both photobioreactor geometry and algae concentration need to be considered to achieve improved growth conditions for algae cultures.

### Spectral conversion materials for microalgae cultivation in open ponds

**Fig. 10:**
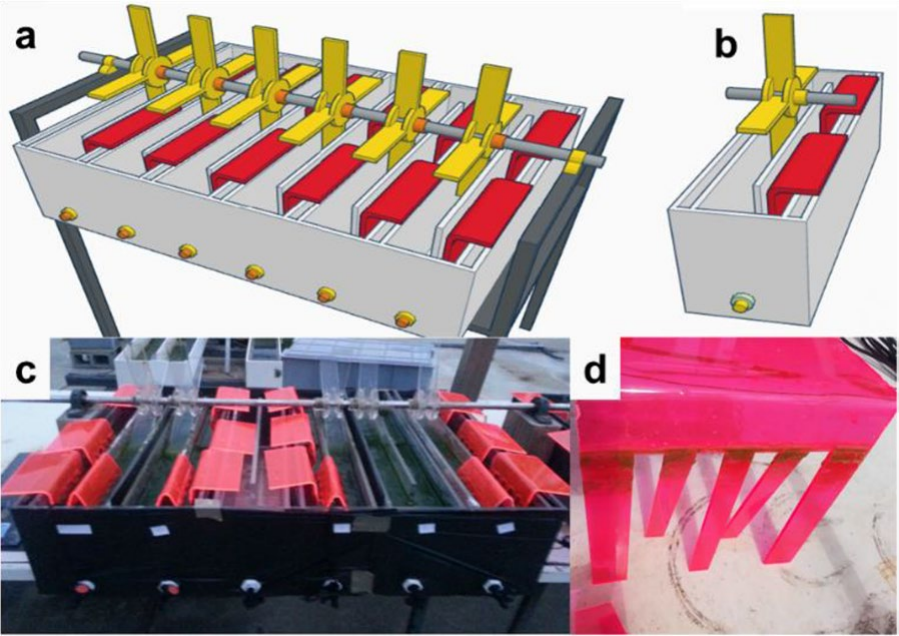
**a** Schematic, **b** singular, and **c** structure view of raceway ponds equipped with red spectral conversion panels with a culture volume of 21.5 L for each pond. **d** *Scenedesmus sp.* biofouling on a panel during 15 days of the cultivation period. Reproduced with permission from [137].

Circular ponds, tanks, and raceway ponds are the most widely used open pond systems [133, 134]. Compared to closed photobioreactors, the main advantage of open ponds includes easy construction and operation. The major constraint, however, is poor light utilization by microalgae in open ponds [135]. Considering the operational depth greater than 20 cm for large-scale microalgae cultivations and light can only penetrate the top few centimeters of the culture, only 15–20% of the microalgal cultures receive sufficient light for photosynthesis [136]. As a result, photolimitation remains one of the main bottlenecks for microalgal cultivation, resulting in rather low biomass productivity. Hence, light delivering systems have been proposed as a potential method to increase the availability of light to microalgal cells in open ponds. For example, Moheimani et al*.* [137] developed a red luminescent solar concentrator (LSC) for microalgae cultivations in outdoor raceway ponds, as shown in Fig. 10. These red LSCs convert the majority of high energy photons of the solar spectrum to red photons but are largely transparent to infra-red. More importantly, the red LSCs deliver the fluorescently generated light into the depth of microalgal cultures in raceway ponds. Outdoor experiments demonstrated an increase in biomass productivity and phycocyanin productivity of *Arthrospira platensis* respectively by 26% and 44%. The reliability of using the developed light delivering system for increasing biomass productivity in open ponds is further examined by the same group with an emphasis on various algae species and growth environments [138]. Biomass productivity of *Scenedesmus sp.* significantly increased by 18.5% and protein, lipid, and carbohydrate productivity of Scenedesmus sp. were also improved by 35%, 20%, and 16% when red LSCs used. Even in anaerobically digested food effluent having high turbidity in open ponds, red LSCs can significantly increase biomass productivity and nitrogen assimilation of a *Chlorella sp.* and *Scenedesmus sp.* consortium culture [139].

**Fig. 11:**
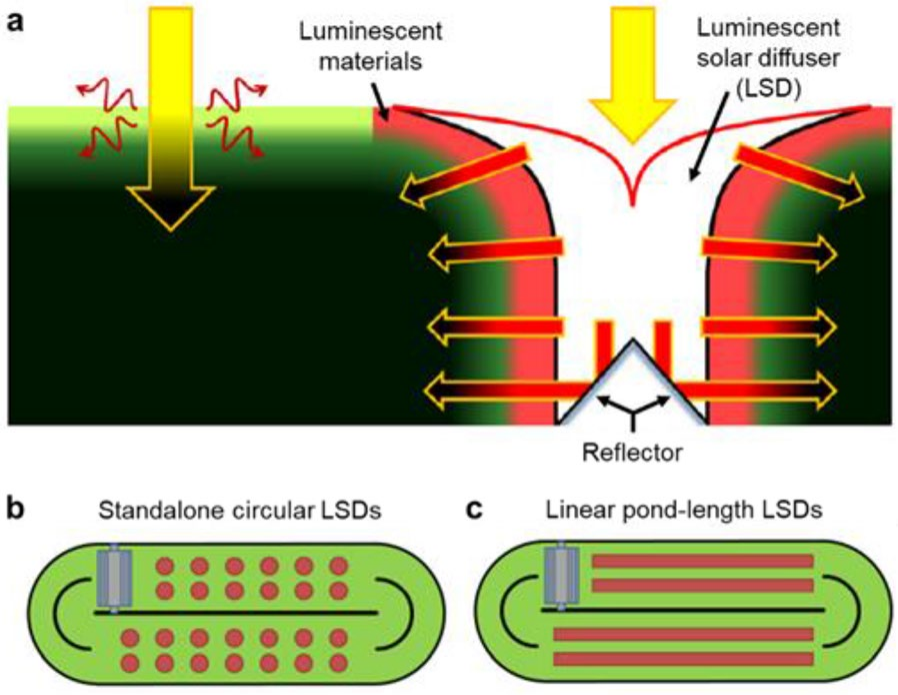
**a** Cross-section schematic of a luminescent solar diffuser (LSD) to mitigate low penetration of incident light in open ponds for microalgae cultures. **b**,** c** Two ways of LSDs used in open ponds: standalone circular devices (**b**) or a linear array (**c**). Reproduced with permission from [140].

Videira et al*.*, on the other hand, designed a luminescent solar diffuser (LSD) as a light distributor [140]. The LSD takes advantage of luminescent materials, collects the impinging light, spectrally converts it into more photosynthetically effective light, and then delivers the converted light through the depth of the pond for algae. The diffuser can be made of transparent polymer material, particularly polyethylene which simply floats due to its low density (< 1 g/cm^3^). A thin luminescent layer is also coated on the diffuser for spectral conversion (Fig. 11a). The generated luminescent emissions can be efficiently guided into algae cultures through LSDs mainly because of the lower refractive index mismatching between the LSD/algae culture (1.5:1.3) interface than that between the LSD/air (1.5:1.0). In addition, the luminescent emitted photons could be partly recycled due to the existence of the reflecting cone at the bottom of the LSD. For practical applications in open ponds, the LSDs can be installed either in the form of standalone devices (Fig. 11b) uniformly distributed in the ponds or a linear funnel along the length of the pond (Fig. 11c). The diffuser collects the impinging light, spectrally converts it into a more useful wavelength, and then delivers the converted light through the depth of the pond for algae. While the absence of experimental demonstration, the calculated data show that the growth rate of algae can be increased by 57% at 1 sun, 50.2% at 0.6 sun, and 35.2% and 0.3 sun intensities by using the luminescent solar diffuser. However, the biggest potential disadvantage of using these spectral conversion materials when they are immersed in microalga cultures is the biofouling of the LSDs. Current technologies in anti-biofouling might provide solutions [141, 142].

## Perspectives

**Fig. 12:**
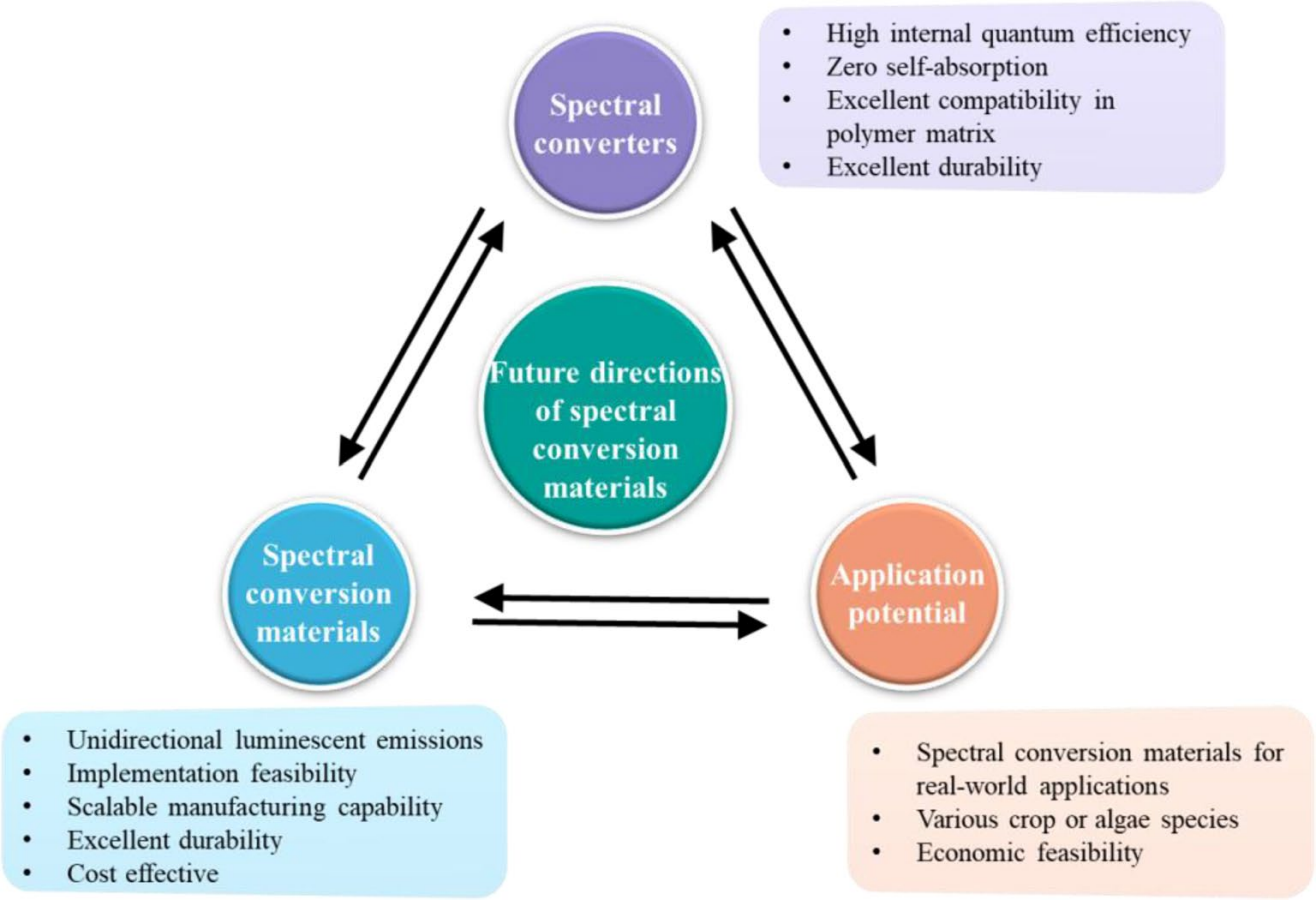
Recommendations for future directions of spectral conversion materials to enhance photosynthesis

Spectral conversion materials have recently been attracting much attention [143, 144] because of the rapid development of numerous novel materials and their consequent versatile applications. To make a successful spectral conversion material, precise spectral management and high efficiency are all equally important since the photosynthetic organisms use the narrow spectrum more efficiently than the broad spectrum of light [145]. Research groups nowadays mainly focus on three directions, as shown in Fig. 12, highly efficient spectral converters, rational material design, and their application potential.

1. Regarding the spectral converters, the target is to simultaneously achieve high internal quantum efficiency and near-zero re-absorption efficiency. The design of semiconductor nanocrystals [146], rare earth complexes [147] and phosphors [148] enables control in Stokes shifts (that is, a separation between absorption and emission peak) and has been found to successfully decrease the re-absorption by the converters themselves while maintaining their high internal quantum efficiency. In addition, the ideal converters must have excellent compatibility, especially in a polymeric matrix, and outstanding durability for long-term applications outdoors.
2. In terms of spectral conversion materials themselves, the goal of research on architecture designs is to increase their overall efficiency. The key is to address the light-trapping of the internally generated light in such materials. The potential methods include either the weakening of total internal reflection by decreasing the refractive index mismatch between the spectral conversion materials and the surrounding environment, or the recycling of the generated photons to increase the emission directionally. Only very few studies, however, have been examined to increase the overall efficiency of the spectral conversion materials for greenhouse claddings by employing unidirectional light-extracting photonic microstructures. For practical applications, these light-extracting photonic microstructures must be easy-to-fabricated and cost-effective.
3. For application potential, further studies must be performed with a specific emphasis on the technology transition from laboratory to real-world application. Several key questions must be answered when it comes to the widespread adoption of this technology, including the impact of spectral conversion materials on photosynthesis across the huge diversity of crop or algae species, weather resistance properties of such materials, and the economic feasibility.

## Summary

Photosynthesis is the basis of both algae and plant growth and improving photosynthesis can contribute toward greater food security and alleviating energy crises with the increasing world population. Compared to photon management with artificial lighting, the use of spectral conversion materials through a photoluminescent process has demonstrated a viable and sustainable means of improving the sunlight utilization efficiency for photosynthetic organisms. In this review, we systematically discuss the spectral conversion materials from photoluminescent converters and fundamental photonics designs to their reported applications. Even though some successful applications in promoting photosynthesis of both plants and microalgae, there remain a number of challenges in the field. These challenges include the development of high-quality luminescent converters, the breakthrough in photonic designs to improve the overall efficiency of the spectral conversion materials, and the application potential of such materials in the real world. In one word, spectral conversion materials promise an effective photon managing way for efficient applications in greenhouses, algae cultivation systems, and other protected environments and have the potential to increase photosynthesis and biomass production.

## Data Availability

Not applicable.

## Abbreviations

PAR: Photosynthetically active radiation
TIR: Total internal reflection
PPFD: Photosynthetic photon flux density
LSCs: Luminescent solar concentrators
PVs: Photovoltaics
QDs: Quantum dots
PMMA: Polymethylmethacrylate
LSD: Luminescent solar diffuser
LED: Light-emitting diode
